# Effects of the Hidden Curriculum in Medical Education: Scoping Review

**DOI:** 10.2196/68481

**Published:** 2025-09-15

**Authors:** Sebastian Parra Larrotta, Erwin Hernando Hernández Rincón, Daniela Niño Correa, Claudia Liliana Jaimes Peñuela, Alvaro Enrique Romero Tapia

**Affiliations:** 1 School of Medicine Universidad de La Sabana Chia Colombia; 2 Department of Family Medicine and Public Health School of Medicine Universidad de la Sabana Chia Colombia; 3 Department of Mental Health and Psychiatry School of Medicine Universidad de La Sabana Chía Colombia

**Keywords:** hidden curriculum, medical education, professionalism, concept formation, professional ethics, humanism

## Abstract

**Background:**

Medical education now focuses on developing skilled and dependable professionals, with particular attention to the hidden curriculum and its influence on professionalism and humanism.

**Objective:**

This scoping review aimed to analyze the available evidence on the benefits and adverse effects of the hidden curriculum in medical education.

**Methods:**

A scoping review of the literature available in the indexed databases PubMed, Scopus, ScienceDirect, and Latin American and Caribbean Health Sciences Literature (LILACS) with MeSH (Medical Subject Headings) descriptors was conducted on the effects of the hidden curriculum in medical education between January 2000 and April 2024. A total of 29 papers were selected for the review.

**Results:**

Our review included studies from 10 countries, most of which were descriptive and cross-sectional, revealing both positive and negative impacts of the hidden curriculum in medical education. These include the transmission of implicit values and the influence on forming skills and professional identity. It was found that some elements contributed to the integral development of students, and others generated challenges that affected the quality of medical education. Likewise, the need for further research to design implementation strategies in different medical schools was described.

**Conclusions:**

The hidden curriculum proves to have both a positive and negative impact on the attitudes and values of medical students. The findings highlight the need to generate greater awareness and proactive strategies in educational institutions to improve the quality of training and promote the holistic development of future health professionals.

## Introduction

### Background

Over the years, medical education has undergone essential transformations to train skilled and reliable professionals who promote health in all people without forgetting the humanistic attitude that the profession demands [1]. In response, medical schools have committed themselves to building a curriculum that allows them to provide society with physicians who respond adequately to the population’s health needs and who are efficient in the practice of their profession [2].

Thus, the curriculum has been the subject of research, where it has been explored in such a way that more than one type of curriculum has been found within the educational process [3]. This has led to the recognition that medical education is a cultural process influenced by external forces, which is how, in 1968, the term ‘hidden curriculum’ gained importance. Jackson [4] described it as “the tacit ways in which knowledge and behavior are constructed, outside the formally programmed courses and subjects” [5]. A study by Hafferty and O’Donnell [6] first documented the hidden curriculum phenomenon in medical education by observing how students develop their professional identity through hidden curriculum instead of formal learning experiences. According to Hafferty and O’Donnell [6], “Hidden curriculum are the customs, rituals, and taken-for-granted aspects of education in the health professions, particularly those that learners experience during interactions with faculty and clinicians in practice settings.” In contrast to formal and null curricula with clear rules and expectations, the hidden curriculum is rarely planned or stated. Through the use of hidden curriculum, instructors were able to shape students’ ideas about the significance and applicability of patients’ knowledge. During this process, learning is generated with no apparent relation to what has been previously established, which is why it can be seen as not very significant; however, evidence shows that it plays an essential role in the fulfillment of educational goals and that it should be included in academic training programs [6,7].

The term hidden curriculum is frequently, though inaccurately, used interchangeably with informal or implicit curriculum; however, these terms have distinct conceptual meanings in medical education literature. Lawrence et al [8] describe the hidden curriculum as the implicit influences arising specifically from institutional culture, organizational structure, and social interactions, significantly shaping professional development and medical identity. In contrast, the informal curriculum refers to spontaneous, unstructured learning that occurs without formal planning, either inside or outside formal educational environments. Meanwhile, the implicit curriculum includes educational content integrated implicitly into institutional practices and expectations, though never explicitly documented [8]. Clearly distinguishing these terms is essential, as the unique nature of the hidden curriculum presents particular methodological challenges for objective measurement and evaluation of its impacts. To facilitate understanding and conceptual differentiation among hidden, informal, and implicit curricula, Table 1 summarizes their key characteristics and provides illustrative examples within medical education.

**Table 1:** Differentiating hidden, informal, and implicit curricula in medical education^a^.

Curriculum type	Definition and key concept	Examples
Hidden	Implicit influences arising from institutional culture, organizational structure, and social interactions	Implicit hierarchy among medical specialties and gender biases in clinical practice
Informal	Spontaneous, unstructured learning experiences occurring without formal planning	Informal discussions among students and faculty and spontaneous clinical discussions in corridors or cafeterias
Implicit	Educational content implicitly integrated into institutional practices, not formally documented	Tacit expectations of professionalism and implicitly assumed ethical behaviors in clinical practice

^a^Adapted from the study by Lawrence et al [8].

Considering the points mentioned previously in the text, it is known that some skills, behaviors, and values inherent to the medical profession are learned through the hidden curriculum [9]. Empathy, respect, confidentiality, and care are among the values learned through experiences in clinical settings. In the hospital setting, the medical community faces challenges ranging from supervised and respectful knowledge construction to witnessing hierarchy, mistreatment, and exploitation by teaching and care staff during their academic process [10].

### Objectives

The primary objective of this review was to conduct a comprehensive analysis of the available evidence regarding the impact of the hidden curriculum in medical education, examining both its benefits and adverse effects. This includes assessing how informal learning experiences, unstructured interactions, and observed behaviors within clinical and educational environments can positively influence the professional and personal development of medical students. In addition, the review aims to investigate potential negative outcomes, such as the perpetuation of undesirable values or stereotypes, and their impact on educational quality and student well-being.

Despite the evident impact of the hidden curriculum, much of the existing research focuses primarily on formal teaching practices, neglecting the hidden dimensions of medical education. As a result, this literature remains scattered and fails to provide a comprehensive analysis of the impact of the hidden curriculum in shaping medical education.

This study aims to address that gap by focusing on specific research questions:

- What are the main effects of the hidden curriculum on medical training, both positive and negative?
- What strategies have been proposed or implemented to mitigate the negative effects of the hidden curriculum in medical education?

By showcasing the varied importance of the hidden curriculum across different medical schools, the review seeks to provide evidence that will encourage other institutions to recognize and incorporate the hidden curriculum into their learning objectives and goals. Ultimately, the review aims to offer practical recommendations for integrating the hidden curriculum in a way that enhances overall medical education and better prepares future health care professionals.

## Methods

### Ethical Considerations

Written informed consent and ethics approval were not required due to the nature of the study.

### Study Design

The PRISMA-ScR (Preferred Reporting Items for Systematic Reviews and Meta-Analyses extension for Scoping Reviews) statement was used to perform this scoping review.

### Eligibility Criteria

This review primarily included qualitative studies with an interpretative perspective, featuring articles that used thematic analysis and grounded theory methodology, as well as opinion pieces, systematic reviews, scoping reviews, and literature reviews. The study excluded quantitative research, focusing instead on gaining deeper insights and understanding the nuanced impact of the hidden curriculum rather than measuring it statistically. Editorial comments and letters to the editor were excluded. Eligible participants were undergraduate and postgraduate medical students. Studies involving other health professions, such as nursing, pharmacy, nutrition, dentistry, and psychology, were not included. Articles included those published between 2000 and April 2024, with full-text availability, whose titles or abstracts incorporated research on the effect of the hidden curriculum in medical education, including values such as professionalism, medical ethics, and humanism. The 2000 to 2024 timeframe was chosen to capture the evolution of research on the hidden curriculum in medical education. Other curricula different than the hidden curriculum were excluded. We included postgraduate students in residency programs such as anesthesia, surgery, family medicine, emergency medicine, and radiology because of their relevance to study objectives and the impact of the hidden curriculum within these fields.

### Information Sources

The PubMed, Scopus, ScienceDirect, and Latin American and Caribbean Health Sciences Literature (LILACS) databases were systematically searched for literature published between January 2000 and April 2024.

### Search Strategy

The search strategy followed systematic search principles and was guided by the population, intervention, comparison, and outcome (PICO) framework. The search terms included the keywords “Education,” “Medical,” “Curriculum,” and “Professionalism” using the Boolean operator AND to obtain results for the search query “Medical Education” [MeSH] AND “Hidden Curriculum” [MeSH] AND “Professionalism” [Mesh].

Experts were not formally consulted during the development of the search strategy. Only qualitative studies published between 2000 and 2024 were eligible for inclusion in this review. The search was limited to studies published in English, Spanish, or Portuguese. The review did not use a standardized quality assessment tool, as its primary aim was to map existing research rather than conduct a detailed critical assessment of individual studies.

### Selection of Sources of Evidence

Two authors (SPL and DNC) screened the titles and abstracts of identified studies based on the inclusion and exclusion criteria. The full texts of the shortlisted studies were analyzed and evaluated independently for eligibility by the same 2 authors (SPL and DNC). In instances of uncertainty, the other 3 authors (EHHR, CLJP, and ART) were consulted, and decisions were made by consensus.

### Data Charting Process (Data Extraction)

Two reviewers (SPL and DN) independently gathered relevant data from the articles included in the review. These data covered several aspects: title, article characteristics (eg, the publication year, country, and field of education), participant details (eg, educational degree and medical residency), and specifics related to the intervention (eg, actual impact and future outcomes in professional skills). Any disagreements between the reviewers were resolved through consultation with the other 3 authors (EHHR, CLJP, and ART), and decisions were reached by consensus.

### Data Items

Articles were included if they featured any independent variable related to the following: presence and characteristics of the hidden curriculum (ie, implicit values, behaviors, and attitudes within medical education settings), educational contexts (ie, academic institutions and hospital settings), and educational stages (ie, undergraduate and postgraduate).

## Results

### Study Selection

**Figure 1:**
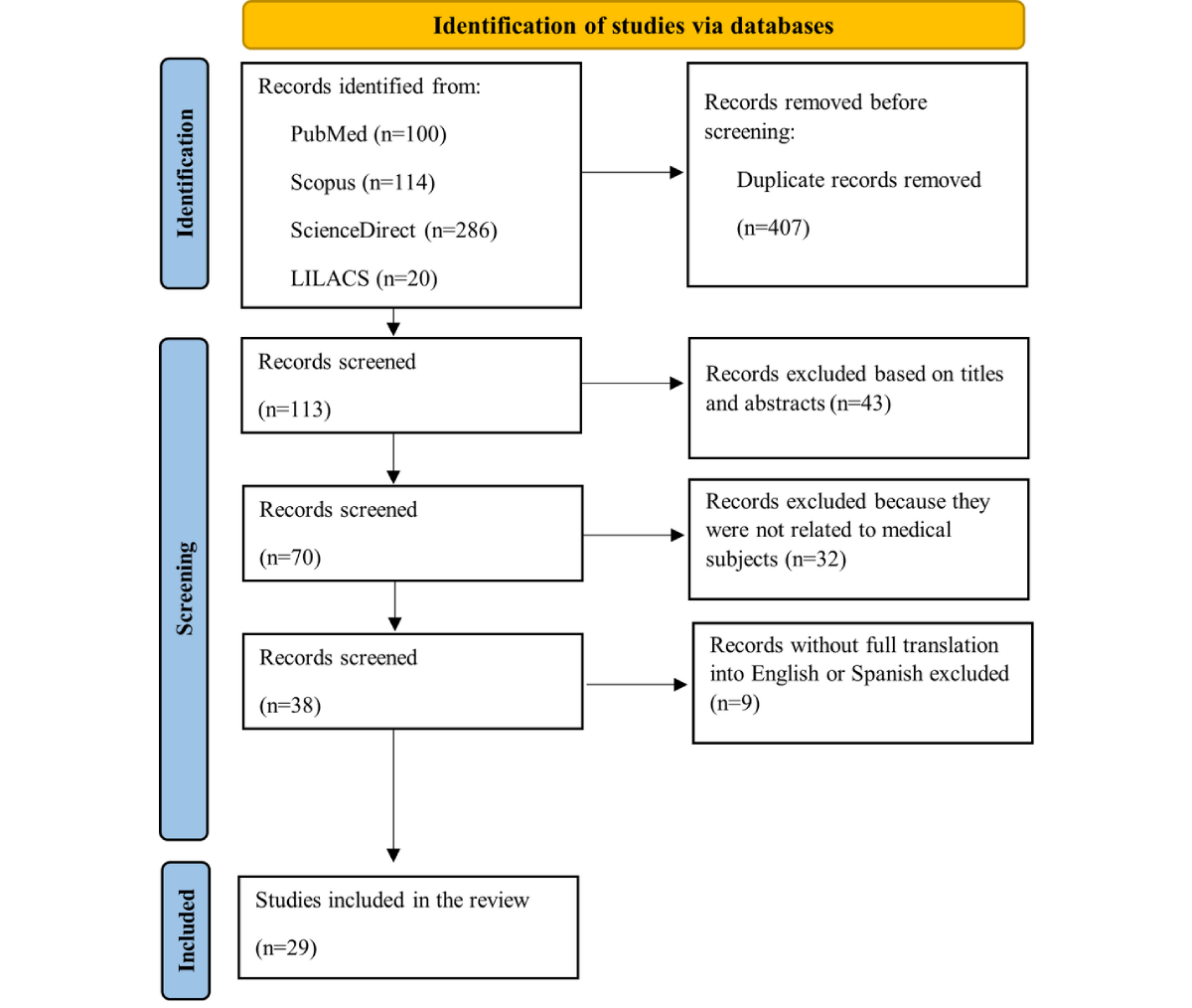
PRISMA (Preferred Reporting Items for Systematic Reviews and Meta-Analyses) 2020 flow diagram for new systematic review that include searches of databases and registers only.

A total of 520 documents were identified in the databases through an electronic literature search, including 100 from PubMed, 114 from Scopus, 286 from ScienceDirect, and 20 from LILACS. After duplicates were removed, 113 articles were screened. The initial title and abstract screening excluded 43 articles, leaving 70. Of these, 32 focused on health areas other than medicine, and 9 lacked full translation into English or Spanish. Excluding these 41 articles left 29 for inclusion in the qualitative synthesis of the scoping review (Figure 1).

### Study Characteristics

The characteristics of all included studies are summarized in Multimedia Appendix 1 [11-39]. Publication dates ranged from 2007 to 2024. Most studies were conducted in the United States (14/29,48%) [11,16-20,24,25,28,31,33,34,38,39], followed by the United Kingdom (4/29, 14%) [12,13,36,37], Iran (3/29, 10%) [14,15,35], Brazil (2/29, 7%) [29,32], Canada (1/29, 3%) [22], Sweden (1/29, 3%) [21], Ireland (1/29, 3%) [23], the Netherlands (1/29, 3%) [27], Israel (1/29, 3%) [26], and Argentina (1/29, 3%) [30]. In terms of methodology, qualitative studies were most common (15/29, 52%), using different designs such as thematic analysis and grounded theory (6/29, 21%) [12,13,15,24,26,29], evaluation design (3/29, 10%) [17,19,33], exploratory and observational approaches (3/29, 10%) [14,23,30], intervention studies (2/29, 7%) [16,33], and discourse analysis (1/29, 3%) [34]; other study types included literature reviews (3/29, 10%) [20,27,33], mixed methods (2/29, 7%) [18,28], systematic and scoping reviews (3/29, 10%) [21,25,35], a position paper (1/29, 3%) [11], integrative reviews (2/29, 7%) [32,36], an innovation report (1/29, 3%) [38], a retrospective study (1/29, 3%) [31], and a perspective article (1/29, 3%) [39].

### Synthesis of Results

In terms of content, the articles included in this scoping review revealed different benefits [13-25] and adverse effects [26-31] of the hidden curriculum present in medical education during different moments of academic training, such as undergraduate [8,10,11,13-19,24-31] and postgraduate education [20-23], as well as tools and strategies for its implementation within the learning objectives and goals of different medical schools for the development of professional skills in medical personnel in training [11,33-37]. Similarly, 3 categories were considered for the analysis of the available literature: “benefits of the hidden curriculum in medical education,” “negative effects of the hidden curriculum in medical education,” and “implementation strategies and limitations within the medical education process.”

### Categorization and Frequency of Effects of the Hidden Curriculum

The effects of the hidden curriculum were categorized and quantified into 2 primary domains: positive (beneficial) and negative (adverse). Among the 29 studies analyzed, (27/29, 93%) reported at least one positive outcome, including the development of professional identity (12/29, 41%), ethical and moral reasoning (7/29, 24%), empathy and humanistic values (5/29, 17%), and reflective thinking (4/29, 14%). Meanwhile, (17/29, 59%) described adverse effects, such as reinforcement of medical hierarchies (6/29, 21%), emotional detachment or stress (5/29, 17%), and value incongruence between formal and informal teachings (5/29, 17%). A detailed summary of these categorizations is presented in Table 2. In this process, multiple studies reported more than one effect.

**Table 2:** Categorization and frequency of effects of the hidden curriculum (N=29).

Effect category and subcategory			Studies, n (%)		References
**Positive^a^**					
	Professional identity formation	12 (41)		[11-13,16,18,20,26,29,30,32,34,37]	
	Ethical and moral development	7 (24)		[11,12,15,20,25,32,37]	
	Empathy and humanistic values	5 (17)		[13,24,25,31,36]	
	Reflective thinking and critical judgment	4 (14)		[12,17,18,23]	
**Negative^b^**					
	Reinforcement of hierarchy or authoritarianism	6 (21)		[10,19,20,22,28,33]	
	Emotional stress and detachment	5 (17)		[13,16,17,23,31]	
	Incongruence between taught and modeled values	5 (17)		[11,21,26,33,37]	

^a^Categories are not mutually exclusive; some studies contributed to more than one subtheme.

^b^Seventeen of the 29 studies reported at least one adverse effect. The subcategories listed capture most of these, although some studies contributed negative aspects not easily classified under the three main themes.

## Discussion

### Principal Findings

This scoping review investigated the role of the hidden curriculum in medical education and its positive and negative aspects. The results highlight critical insights: one of the key findings of this study is that the hidden curriculum is an unavoidable, powerful force that shapes how medical trainees develop their professional identities, medical professionalism, and humanism. The findings of this study also show that a hidden curriculum can effectively strengthen the physician-patient relationship. The hidden curriculum proved to be the dominant factor that determines professional adaptability for medical practitioners who face ongoing changes in clinical practice. Medical students learn essential professional attitudes and behaviors through the pervasive influences of the hidden curriculum. The hidden education system surpasses traditional classroom learning because it shapes students’ moral values and ethical conduct as well as their approach to making important decisions. This review also suggests that medical institutions should create proactive approaches and refine procedures for an implicit hidden curriculum that affects medical professionals.

### Comparison With Prior Literature

**Figure 2:**
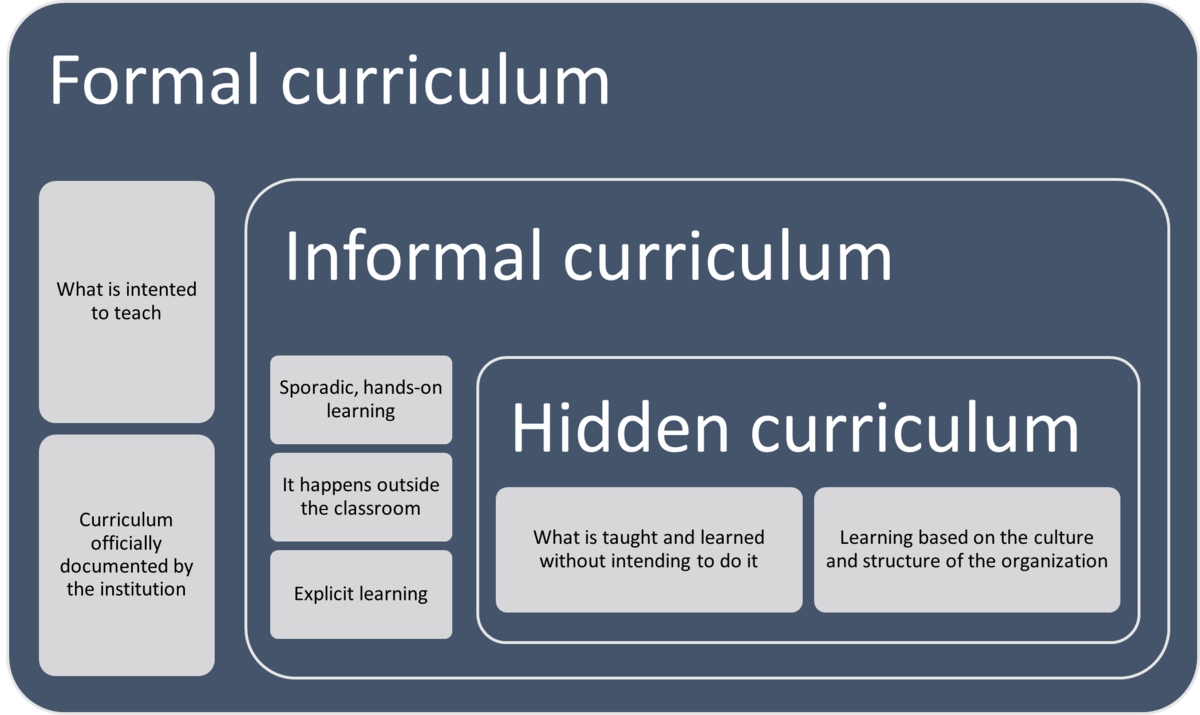
Types of curricula described in the literature (adapted from the studies by Hafferty and O'Donnell [6] and Hafferty [10]).

The review aligns with the existing research showing how the hidden curriculum’s power remains unrecognized in its ability to mold medical student experiences. The first description of the hidden curriculum was made in 1970 by Jackson [5]; however, it was not until 1994 that its relationship with medical education was defined by Hafferty [10], who stated that the training and learning process of the medical profession was influenced by cultural aspects and by the context in which the students developed and attributed to the hidden curriculum the reason for most of their mistakes [7]. Culture significantly shapes the hidden curriculum by influencing medical students to incorporate professional standards and core values through educational settings that differ across societies. Some cultural influences encourage patient-centered care and professionalism, but other cultural elements maintain hierarchical structures while promoting discrimination and emotional suppression. Likewise, learning has been approached through 3 dimensions of the curriculum, which are represented in Figure 2 [6,10]: the formal curriculum, what is documented to offer; the informal curriculum, how the curriculum is carried out through the interactions of the community; and the hidden curriculum, all that learning that occurs outside the expected and stipulated [11], which is where skills that cannot be taught through the formal curriculum are developed.

Given the previously mentioned positive and negative effects, several authors in the analyzed literature explore the hidden curriculum. They highlight both the benefits and drawbacks of the hidden curriculum in medical training, where values such as empathy, respect, and professionalism are cultivated. Nevertheless, even the physician-patient relationship can be affected by negative interactions and experiences in clinical practice settings [12].

However, this review expands on previous research by highlighting the benefits of the hidden curriculum. First, a benefit is described in the search, construction, and strengthening of professional identity [13]. When students face negative experiences and behaviors in the hospital environment, it is necessary to confront their own identity with the one they are expected to acquire, which generates a reflection about the professionals they wish to become in the future. The hidden curriculum gives students the skills they need to adjust to different professional situations in addition to helping them establish their identities. The development of critical soft skills, including empathy, teamwork, and communication, is positively impacted by the hidden curriculum [13]. Medical students who master the interpretation and understanding of the hidden curriculum become better prepared for dynamic health care environments, demonstrating greater resilience, innovative problem-solving, and a proactive commitment to lifelong learning [18].

This review finding aligns with the findings by Azmand et al [14] that a hidden curriculum can effectively strengthen physician-patient relationships, where the role models of teachers motivate them to dedicate adequate time to patients, recognizing their concerns beyond their medical conditions and needs. Physician-patient relationships embedded within the hidden curriculum substantially enhance patient outcomes. The hidden curriculum enhances formal medical education by incorporating patient-centered approaches into medical students’ everyday lives, which eventually helps both practitioners and the patients they treat. These relationships were characterized by a sense of ethics, responsibility toward others, and the medical environment [15,16].

By contrast, the influence of the hidden curriculum on the construction of professionalism and the trust that patients and their families place in training professionals has been explored [17]. Teachers and students emphasized that the formal curriculum does not teach them the necessary skills to interact in a dynamic environment such as a hospital and that lived experiences allow them to develop skills and attitudes that are inherent to the medical profession, among which respect, responsibility, compassion, and communication, among others, stand out [18]. The hidden curriculum promotes the value of moral decision-making, transparency, and confidentiality, all of which help to foster trust with patients as well as colleagues. The hidden curriculum is evident at all stages of training [19], from undergraduate to postgraduate programs such as general surgery [20], family medicine [21], radiology [22], and anesthesia [23].

Finally, regarding its benefits, the hidden curriculum has been shown to influence dimensions such as religion, spirituality [24], and humanism [25]. Through these experiences, students developed coping strategies for emotional stress when treating patients facing serious illness, family difficulties, or death. They also acquired values such as empathy, communication, respect, and emotional stability—skills learned through real-world practice rather than the formal curriculum.

Through a scoping review of the literature, it was determined that the available evidence, to a great extent, revealed the adverse effects of the hidden curriculum in medical education. Mistreatment [26] and hierarchical dynamics toward students and residents were among the most frequently reported issues, often linked to decreased motivation and commitment to the training process [27]. The students mentioned that 2 different teachers in the same service could improve or affect the learning environment and their performance. Thus, mistreatment by colleagues and teachers during the selection process of medical-surgical specialties has even been described [28], which negatively impacts their decisions. Studies link mistreatment exposure to higher stress levels and burnout symptoms, along with diminished empathy, which harms both the students’ mental state and their competency in providing patient care [28].

In addition, another component of the hidden curriculum that generates adverse effects is the burden and pressure on undergraduate and graduate students [29]. They explained that they were required to respond in an accelerated manner to their obligations to their patients, which affected their care in terms of time and quality. This attitude of immediacy in practice scenarios is often generated in teachers by a conflict between the teaching role and the caring role [30], in which, by neglecting one or appropriating the other, attention and respect for the patient or their teaching activity with students are affected. The implicit demands of the hidden curriculum may cause stress, which could have a negative impact on students’ general well-being and long-term ability to adjust professionally. Addressing this role conflict is essential, as medical training should prioritize proper practice, integrating clear instruction with comprehensive patient care.

The dual impact of the hidden curriculum on medical education, its capacity to promote humanistic values and simultaneously induce stress, has been recently explored. Auckley et al [31], through their analysis of the Humanism in Medicine Initiative, demonstrate that while extracurricular humanism-focused activities significantly enhance student well-being and professional identity, moderate engagement paradoxically correlates with increased stress. Such findings underline the nuanced role of the hidden curriculum in shaping medical students’ experiences and highlight the need for balanced engagement.

Regarding humanism, although some studies highlight the benefit of the hidden curriculum in integrating these qualities with medical professionalism, others suggest a disconnect between clinical skills and the humanistic aspects of medical education [32]. Skills such as communication, decision-making, respect, and autonomy are often acquired through implicit learning, which tends to receive less emphasis. This limited focus may reduce the humanistic approach to patient care when these students later practice as physicians [32]. Table 3 compares the positive and negative effects of the hidden curriculum.

**Table 3:** Comparison of the positive and negative effects of the hidden medical education curriculum.

Feature	Positive effects	Negative effects
Professional identity	Consolidation of professional identity through confrontation between personal and expected roles [13]	Imposed identity leading to dissonance and emotional distress [13]
Medical professionalism	Reflection spaces to analyze emotions and promote respect and empathy toward teachers, colleagues, and patients [11,17]	Mistreatment, humiliation, and hierarchy normalized as acceptable cultural patterns [11]
Physician-patient relationship	Recognition of patient concerns and needs beyond disease, fostering trust-based relationships [14-16]	Loss of patient interaction opportunities due to efficiency culture and high workload [29,30]
Wellness and mental health	Promotion of self-care, spirituality, and mental well-being [24]	Clinical distress, burnout, low well-being, and depression affecting performance [11]
Humanism	Adoption of communication, empathy, respect, and autonomy through the hidden curriculum [25]	Dehumanization in medical education and patient care perpetuated across generations [11]

### Implementation Strategies and Challenges in Addressing the Hidden Curriculum

To date, the benefits and adverse effects of the hidden curriculum in medical education have been analyzed. This leads to identifying strategies to enhance the virtues and mitigate the unfavorable effects it may have on medical students and the limitations of its implementation. First, studies highlight the importance of constantly evaluating the learning environments in educational institutions and clinical practice scenarios [33,34] so that they become a safe and adequate environment for the acquisition of professional and personal skills for patient care, recognizing that the educational process is based not only on the knowledge to be acquired but also on multicultural education that strengthens professional identity and ethics [34].

Likewise, the importance of strengthening institutional culture in practice scenarios has been described based on humanistic attitudes, such as compassion, curiosity, respect, and empathy, enabling students to identify with these values and carry them into their professional practice [11]. This process should involve the student in situations where they must make ethically complex decisions and face emotions that impact them, supported by teacher guidance and opportunities for reflection [11].

Moreover, recent literature highlights specific interventions aimed at mitigating negative impacts and optimizing the hidden curriculum. Hosseini et al [35] identify key strategies, such as the implementation of new curricula, team-based clinical clerkships, and longitudinal faculty development workshops. These strategies are essential to manage the implicit values and unintended messages conveyed through informal learning experiences and interactions, reducing the adverse outcomes associated with the hidden curriculum.

In addition, Howick et al [36] propose a transformative approach to leverage the hidden curriculum as a tool for fostering empathy among medical students. They suggest evidence-based interventions, such as early patient exposure, improving clinical environments, and explicit empathy education as critical components of what they term an “empathic hidden curriculum.” Such approaches are crucial in countering empathy erosion frequently observed during medical training.

**Figure 3:**
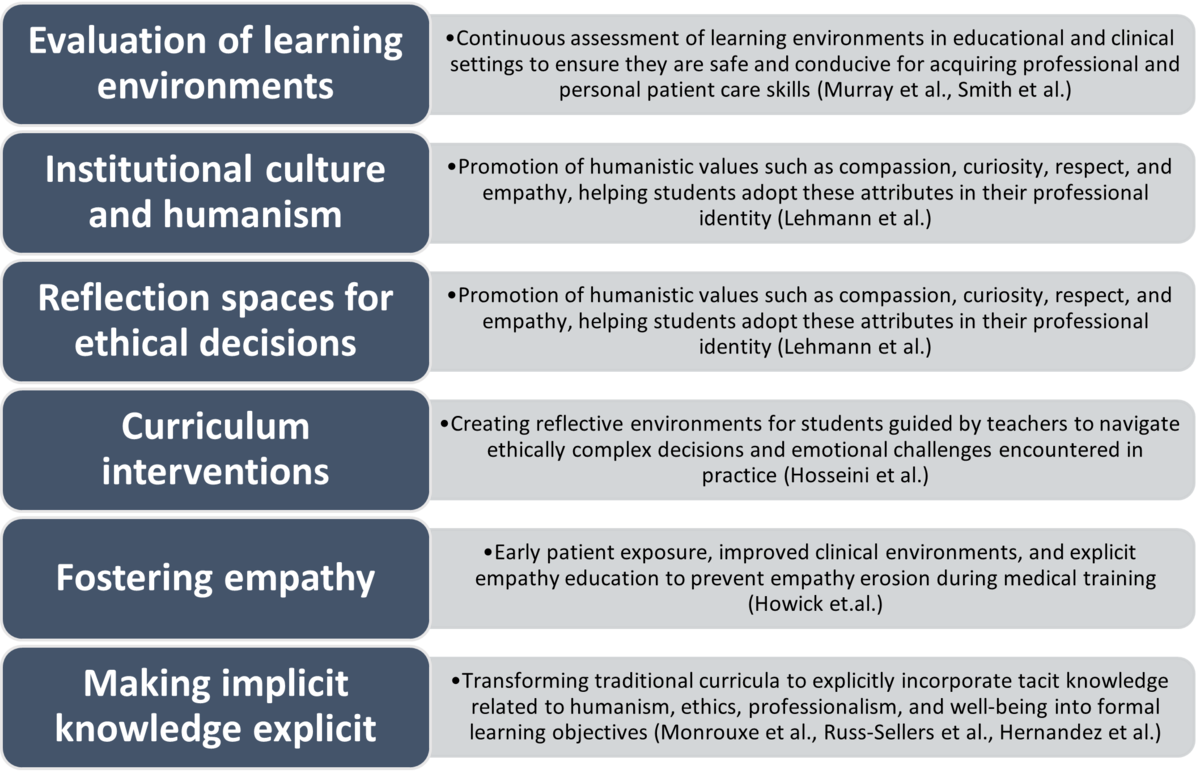
Hidden curriculum implementation strategies and limitations in medical education.

Finally, one of the most significant challenges, with several limitations, is to include some of the knowledge acquired through the hidden curriculum within an explicit curricular objective [37]. Educational environments are increasingly aware of the dissociation that exists between what they wish to teach and what their students learn; strategies of early immersion of students with the communities they are going to serve, including an early approach to hospital environments [38] and accompaniment based on humanism, professionalism, and ethics [39], make it possible to put into practice some of the tacit learning and make it visible. The main limitation during this process and a source of future research is the transformation of traditional curriculum and strategies to include aspects of humanism, well-being, and medical professionalism within the learning objectives of future professionals [39]. Some of these strategies are illustrated in Figure 3.

### Implications

The findings of this study have significant implications. Aspects of the hidden curriculum should be incorporated into official educational frameworks by educational policymakers. Universities ought to spend money on extracurricular activities that promote the development of professional skills.

To guide future research, several key recommendations are proposed. First, comparative studies evaluating the effectiveness of different educational approaches, such as integrating the hidden curriculum versus more explicit methods or practical versus theoretical strategies, would be valuable. In addition, incorporating digital interventions could provide insights into how technology can influence medical training, given recent advancements in this area. It is also crucial to consider implementation costs and discuss resource accessibility to enable financial and logistical analyses of proposed interventions. Long-term follow-up studies of students would be beneficial to assess how they apply learned lessons in their clinical practice. This will help determine if medical education achieves its goals of producing competent and ethical professionals and explore whether the hidden curriculum practices result in tangible benefits for patients and improvements in educational quality.

### Strengths

The study’s strength lies in its use of qualitative research methods, which capture detailed perspectives on the hidden curriculum in medical education. The research investigates medical education from 2000 to 2024 to provide a comprehensive analysis of historical and recent developments. The extended timeframe enables researchers to detect both evolving patterns and enduring difficulties that come from the hidden curriculum. The comprehensive range of themes studied across different publications emerges as a key strength in this evaluation. This assessment brings together research from different medical disciplines to demonstrate how the hidden curriculum expresses itself distinctly between specialties, thus advancing our collective understanding of its effects.

### Limitations

This scoping review focused primarily on qualitative studies with interpretative perspectives and excluded quantitative research, which may have limited the breadth of evidence regarding the hidden curriculum’s impact. Certain studies, such as those involving other health professions (eg, nursing and pharmacy), were excluded, potentially omitting relevant insights that could provide a broader understanding of the hidden curriculum’s impact across different fields. The exclusion of Web of Science, Embase, and Google Scholar may have led to the omission of relevant studies, potentially limiting the comprehensiveness of the findings. The review was limited to studies published in English, Spanish, and Portuguese, which might exclude relevant research published in other languages. In addition, the inclusion of only published studies could introduce publication bias, as studies with negative findings may be less likely to be published. One methodological limitation was not using specific Boolean operators, such as NOT, to explicitly differentiate between hidden, informal, and implicit curricula during the literature search, potentially affecting the breadth and specificity of identified studies. While the review discussed strategies for integrating the hidden curriculum into formal curriculum, it did not fully address the practical challenges and barriers that institutions might face when implementing these strategies.

### Conclusions

This scoping review has described various aspects of the hidden curriculum in medical education and its potential consequences. A synthesis of the available literature shows that the hidden curriculum conveys explicit knowledge and has many subtle but significant effects on medical students. From forming attitudes and values to influencing professional identity, the hidden curriculum has emerged as a crucial factor in shaping the educational experience.

As understanding of these effects progresses, the need for greater awareness and reflection within different medical schools is highlighted. This review underscores the importance of proactively addressing the hidden curriculum, recognizing it as an essential component for the holistic development of future health professionals. Implementing strategies to maximize positive aspects and minimize negative aspects could significantly improve the quality of medical education and contribute to future physicians’ personal and professional growth.

## Appendix

Multimedia Appendix 1 Summary of the documents included in the review.

Multimedia Appendix 2 PRISMA-ScR checklist.

## Abbreviations

LILACS: Latin American and Caribbean Health Sciences Literature
PICO: population, intervention, comparison, and outcome
PRISMA-ScR: Preferred Reporting Items for Systematic Reviews and Meta-Analyses extension for Scoping Reviews
