# Supplementary material for: Effects of the Hidden Curriculum in Medical Education: Scoping Review
Source: JMIR Med Educ. 2025 Sep 15;11:e68481. doi: 10.2196/68481 (PMC12481137; doi:10.2196/68481)
Supplement: Multimedia Appendix 1 [file mededu_v11i1e68481_app1.docx]

| **No.** | **Study (year)** | **Country** | **Design** | **Methods** | **Summary** |
| --- | --- | --- | --- | --- | --- |
| 1 | Lehmann et al. [11] (2018) | USA | Position paper | Not Applicable | Correlation between ethics, professionalism, and hidden curriculum as main strategies within medical education, strategies and challenges for implementing the hidden curriculum. |
| 2 | Mossop et al. [12] (2013) | United Kingdom | Qualitative | Focus group  Thematic analysis | Positive and negative contributions of the hidden curriculum within the medical education training process. |
| 3 | Brown et al. [13] (2020) | United Kingdom | Qualitative | Grounded theory  Contructivistic analysis | Relationship between knowledge and different factors that influence the hidden curriculum to improve medical education, identity, and professional transparency. |
| 4 | Azmand et al. [14] (2018) | Iran | Qualitative | Exploratory | Perceptions and experiences of medical students are primary sources for an in-depth understanding of the hidden curriculum and its functioning. |
| 5 | Safari et al. [15] (2020) | Iran | Qualitative | Grounded theory | Identify factors affecting medical ethics to help develop more effective ways to promote medical ethics in medical education. |
| 6 | Shorey et al. [16] (2013) | USA | Qualitative | Intervention | Identity formation, judgment and life lessons within hidden curriculum learning and medical education. |
| 7 | Mackin et al. [17] (2019) | USA | Qualitative: | Evaluation | The hidden curriculum is as a fundamental part of physicians’ professional and emotional growth in training. |
| 8 | Karnieli-Miller et al. [18] (2011) | USA | Qualitative-cuantitative | Mixed | Relationship between medical students' learning experiences in the hidden curriculum and their contribution to professionalism. |
| 9 | Nittur et al. [19] (2017) | USA | Qualitative: | Evaluation | How curriculum structure and the hidden curriculum influence the formation of professionalism in medical students. |
| 10 | Rogers et al. [20] (2012) | USA | Literature review | Not Applicable | Importance of effectively developing and assessing professionalism as part of the hidden curriculum in medical students. |
| 11 | Rothlind et al. [21] (2020) | Sweden | Systematic review | Not Applicable | Hidden curriculum as a beneficial strategy in training residents during their education process. |
| 12 | Van Deven et al. [22] (2013) | Canada | Qualitative | Case study | The hidden curriculum is present in all medical specialties; in radiology, it has been studied as a way for professionals to integrate with their colleagues. |
| 13 | Moran et al. [23] (2019) | Ireland | Qualitative | Observation | The hidden curriculum as a training strategy and coping mechanism within anesthesia medical education. |
| 14 | Balboni et al. [24] (2015) | USA | Qualitative | Grounded theory | Hidden curriculum is a training process based on learning, behaviors, attitudes, and values according to the ideals of the medical profession. |
| 15 | Martimianakis et al. [25] (2015) | USA | Scoping review | Not Applicable | Humanism is a significant component of the hidden curriculum in the medical career. |
| 16 | Karnieli-Miller et al. [26] (2010) | Israel | Qualitative: | Thematic analysis | Hidden curriculum as part of the institutional environment and organizational culture of medical education. |
| 17 | Boer et al. [27] (2020) | Netherlands | Literature review | Not Applicable | How to empower students through the hidden curriculum to strengthen medical education strategies. |
| 18 | Oser et al. [28] (2014) | USA | Longitudinal study | Mixed methods | Mistreatment as a transversal indicator of the hidden curriculum in the clinical experiences and practices of medical education. |
| 19 | Silveira et al. [29] (2019) | Brazil | Qualitative | Thematic analysis | How the hidden curriculum affects medical students’ socialization process in terms of forming their professional identity. |
| 20 | Montesinos et al. [30] (2012) | Argentina | Qualitative: | Prospective-Observational | Physician‒patient relationship and how work experiences influence the outcome of medical education within the hidden curriculum. |
| 21 | Auckley et al[31](2022) | USA | Qualitative | Retrospective | Demonstrate that while extracurricular humanism-focused activities significantly enhance student wellbeing and professional identity, moderate engagement paradoxically correlates with increased stress |
| 22 | Silva et al. [32] (2013) | Brazil | Integrative review | Not Applicable | How the hidden curriculum and medical education impact students' cognitive and emotional overload. |
| 23 | Murray et al. [33] (2008) | USA | Literature review | Not Applicable | How educators can identify previously undiagnosed elements and potential impacts of their institutional curricula. |
| 24 | Smith et al. [34] (2007) | USA | Qualitative: | Implementation and evaluation | Professionalism is the basic structure of the institution. |
| 25 | Hosseini et al. [35](2023) | Iran | Scoping review | Not Applicable | Identify key strategies such as the implementation of new curricula, team-based clinical clerkships, and longitudinal faculty development workshops |
| 26 | Howick et.al. [36] (2024) | United Kingdom | Integrative Review | Not Applicable | Propose a transformative approach to leverage the hidden curriculum as a tool for fostering empathy among medical students |
| 27 | Monrouxe et al. [37] (2011) | United Kingdom | Qualitative: | Discourse analysis | Importance and basis of the hidden curriculum for developing professionalism and ethical and moral identity in medical students. |
| 28 | Russ-Sellers et al. [38] (2017) | USA | Innovation report | Not Applicable | How to promote early clinical exposure and biomedical integration within the hidden curriculum. |
| 29 | Hernandez et al. [39] (2022) | USA | Perspective | Not Applicable | Hidden curriculum influenced by social, cultural, and belief systems embedded in surgical education and training. |
